# Supplementary material for: Bayesian refinement of protein structures and ensembles against SAXS data using molecular dynamics
Source: PLoS Comput Biol. 2017 Oct 18;13(10):e1005800. doi: 10.1371/journal.pcbi.1005800 (PMC5662244; doi:10.1371/journal.pcbi.1005800)
Supplement: S1 Archive — (BZ2) [file pcbi.1005800.s011.tar.bz2 › gromacs_4.62_bayesian_saxs_refi/share/html/online/xpm2ps.html]

xpm2ps


| xpm2ps Main Table of Contents |

**VERSION 4.6  
Sat 19 Jan 2013**

---

### Description

xpm2ps makes a beautiful color plot of an XPixelMap file.
Labels and axis can be displayed, when they are supplied
in the correct matrix format.
Matrix data may be generated by programs such as do\_dssp, g\_rms or
g\_mdmat.

Parameters are set in the .m2p file optionally supplied with
-di. Reasonable defaults are provided. Settings for the y-axis
default to those for the x-axis. Font names have a defaulting hierarchy:
titlefont -> legendfont; titlefont -> (xfont -> yfont -> ytickfont)
-> xtickfont, e.g. setting titlefont sets all fonts, setting xfont
sets yfont, ytickfont and xtickfont.

When no .m2p file is supplied, many settings are taken from
command line options. The most important option is -size,
which sets the size of the whole matrix in postscript units.
This option can be overridden with the -bx and -by
options (and the corresponding parameters in the .m2p file),
which set the size of a single matrix element.

With -f2 a second matrix file can be supplied. Both matrix
files will be read simultaneously and the upper left half of the
first one (-f) is plotted together with the lower right
half of the second one (-f2). The diagonal will contain
values from the matrix file selected with -diag.
Plotting of the diagonal values can be suppressed altogether by
setting -diag to none.
In this case, a new color map will be generated with
a red gradient for negative numbers and a blue for positive.
If the color coding and legend labels of both matrices are identical,
only one legend will be displayed, else two separate legends are
displayed.
With -combine, an alternative operation can be selected
to combine the matrices. The output range is automatically set
to the actual range of the combined matrix. This can be overridden
with -cmin and -cmax.

-title can be set to none to suppress the title, or to
ylabel to show the title in the Y-label position (alongside
the y-axis).

With the -rainbow option, dull grayscale matrices can be turned
into attractive color pictures.

Merged or rainbowed matrices can be written to an XPixelMap file with
the -xpm option.

### Files

| option | filename | type | description |
| --- | --- | --- | --- |
| **-f** | root.xpm | Input | X PixMap compatible matrix file |
| **-f2** | root2.xpm | Input, Opt. | X PixMap compatible matrix file |
| **-di** | ps.m2p | Input, Opt., Lib. | Input file for mat2ps |
| **-do** | out.m2p | Output, Opt. | Input file for mat2ps |
| **-o** | plot.eps | Output, Opt. | Encapsulated PostScript (tm) file |
| **-xpm** | root.xpm | Output, Opt. | X PixMap compatible matrix file |

### Other options

| option | type | default | description |
| --- | --- | --- | --- |
| **-[no]h** | bool | no | Print help info and quit || **-[no]version** | bool | no | Print version info and quit || **-nice** | int | 0 | Set the nicelevel || **-[no]w** | bool | no | View output .xvg, .xpm, .eps and .pdb files || **-[no]frame** | bool | yes | Display frame, ticks, labels, title and legend || **-title** | enum | top | Show title at: top, once, ylabel or none || **-[no]yonce** | bool | no | Show y-label only once || **-legend** | enum | both | Show legend: both, first, second or none || **-diag** | enum | first | Diagonal: first, second or none || **-size** | real | 400 | Horizontal size of the matrix in ps units || **-bx** | real | 0 | Element x-size, overrides -size (also y-size when -by is not set) || **-by** | real | 0 | Element y-size || **-rainbow** | enum | no | Rainbow colors, convert white to: no, blue or red || **-gradient** | vector | 0 0 0 | Re-scale colormap to a smooth gradient from white {1,1,1} to {r,g,b} || **-skip** | int | 1 | only write out every nr-th row and column || **-[no]zeroline** | bool | no | insert line in .xpm matrix where axis label is zero || **-legoffset** | int | 0 | Skip first N colors from .xpm file for the legend || **-combine** | enum | halves | Combine two matrices: halves, add, sub, mult or div || **-cmin** | real | 0 | Minimum for combination output || **-cmax** | real | 0 | Maximum for combination output |

---

http://www.gromacs.org  
gromacs@gromacs.org
